# Recent advances in synthesis and biological activity of triterpenic acylated oximes

**DOI:** 10.1007/s11101-014-9353-5

**Published:** 2014-04-11

**Authors:** Barbara Bednarczyk-Cwynar, Lucjusz Zaprutko

**Affiliations:** Department of Organic Chemistry, Faculty of Pharmacy, Poznan University of Medical Sciences, Grunwaldzka Str. No. 6, 60-780 Poznan, Poland

**Keywords:** Acyl derivatives of triterpenic oximes, Derivatives of triterpenes, Triterpenes, Triterpenic oximes

## Abstract

During the last few decades more and more attention has been paid to triterpenes—a group of compounds with five- or four-ring skeleton and carboxyl, hydroxyl or oxo groups. Triterpenes with unsubstituted C-3 hydroxyl group can be easily transformed into appropriate ketones and then into oximes. The carbonyl group can be created not only from the hydroxyl group at C-3 position, but also at C-2, C-12 or C-28 positions. Several methods of creation of two = NOH groups within one molecule of triterpene are known. There are also known triterpenes with two carbonyl groups, e.g. at C-3 and C-11 positions, which differ in reactivity: among them only C-3 group can be transformed into oxime. A reactive hydroxyimine group can undergo the action of acylating agents, such as carboxylic acids or their derivatives, also the ones with significant pharmacological activity. Acyl derivatives of triterpenic oximes exhibit important pharmacological activity. The biological tests performed with the use of cell cultures inoculated with viruses showed inhibitory activity of some triterpenic acyloximes against type 1 HSV (H7N1), ECHO-6 and HIV-1 viruses. Another acylated oximes derived from triterpenes shown cytotoxic or antiproliferative activity against many lines of cancer cells. In many cases the pharmacological effects of the tested acyloxyiminotriterpenes were comparable to those of appropriate standard drugs. One of the newest application of acyl derivatives of triterpenic oximes is their ability to form organogels.

## Introduction

Natural resources have been used for thousands of years in order to combat human diseases. Over the last decade the interest in natural products and their mechanisms of action has been intensified and now naturally occurring substances play an increasing role in drug discovery and development. Natural compounds are subjected to miscellaneous chemical transformations with the use of different chemicals in order to obtain numerous new derivatives. The semisynthetic compounds obtained as a result of such transformations often exhibit more advantageous pharmacological activity than mother compounds.

The natural triterpenes are created by living tissues of numerous higher plants. As an example, oleanolic acid, one of the most popular triterpenic acid can be isolated from above 1,600 species of plants (Yeung and Che 2009). Triterpenes can be also created by tissues of some fungi, e.g. such as *Ganoderma lucidum* (Smina et al. 2011), *Inonotus obliguus* (Yin et al. 2008) or *Pisolithus tinctorius* (Zamuner et al. 2005). This group of compounds exhibit a wide range of chemical diversity and biological properties. The pharmacological tests proved e.g. anticancer (Braga et al. 2007), antiviral (Pompei et al. 2009), antibacterial (Fontanay et al., 2008), hepatoprotective (Szuster-Ciesielska et al. 2011), cardiovascular, antihyperlipidemic, antioxidant (Somova et al. 2003), anti-inflammatory (Huguet et al. 2000), antiulcer (Queiroga et al. 2000), analgesic and antinociceptive (Oliveira et al. 2005), antidiabetic (Teodoro et al. 2008) and another effects of many triterpenes. Viral and cancer diseases are now one of the greatest problem for humans, because of many complicated mechanisms of action of viruses and cancer agents. For this reason the greatest hopes of doctors and scientists are connected with antiviral and anticancer activity of natural derived compounds, also triterpenes.

From a chemical point of view, the most important triterpenoid structures are oleanane, ursane and lupane triterpenoids and less known triterpenes belong to dammarane, euphane and taraxastane group.

Triterpenes are compounds derived from natural occuring squalene. After different transformations of squalene numerous polycyclic triterpenic structures are formed, mostly with the C-3 hydroxyl group. Often one or more carboxyl and additional hydroxyl groups are present as well. Such combination of polycyclic structure with unsaturated bonds and functional groups makes the possibility to perform miscellaneous chemical transformations leading to numerous new derivatives. Oleanolic acid, one of the broadest distributed triterpenes, is the excellent example of chemical reactivity of C-3 hydroxyl group and further transformations of the obtained product leading to the receiving of numerous derivatives with advantageous pharmacological activities.

**Scheme 1:**
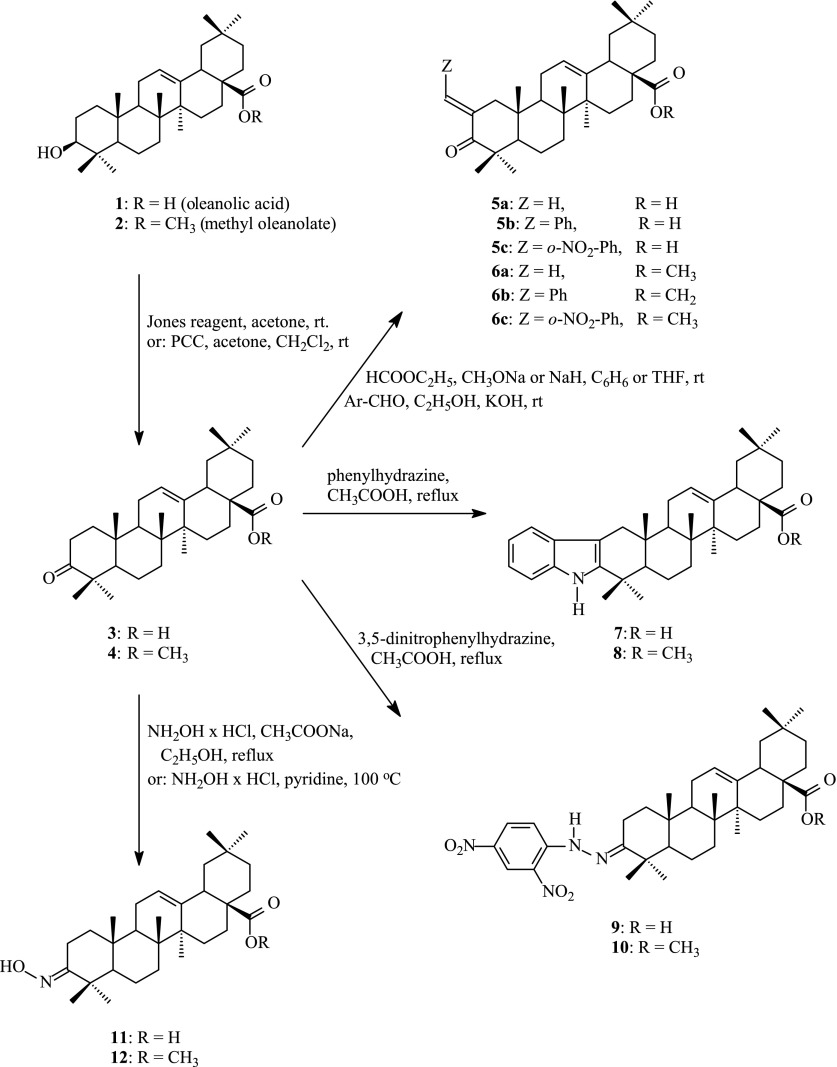
Some important chemical transformations of oleanonic acid (**3**) or its methyl ester (**4**) within A-ring

Hydroxyl group at C-3 position of oleanolic acid (**1**) can be subjected to the reaction of esters synthesis (Scheme 1), e.g. with the use of carboxylic acids, their anhydrides or acyl chlorides (Zhu et al. 2001; Hichri et al. 2003; Ma et al. 2000, 2002 Ali et al. 2002; Chen et al. 2006). The acylation with the appliance of acetic anhydride in pyridine is the general method for reversible protecting of C-3 hydroxyl group, e.g. against the action of oxidizing agents. The alkaline hydrolysis leads to unblocking of this hydroxyl group.

Oleanolic acid (**1**) or its methyl ester (**2**) can be oxidizied with Jones reagent (Chen et al. 2006; Pungitore et al. 2005) or with pyridinium chlorochromate (Ma et al. 2000) to the appropriate 3-oxoderivatives (**3** or **4**, respectively). The above products are known as oleanonic acid (**3**) and methyl oleanonate (**4**), respectively. Formation of reactive C-3 carbonyl function activates the neighbouring methylene group (at C-2 position) so it is possible to obtain many new compounds.

Oleanonic acid **3** and its methyl ester **4** can be subjected to reaction of condensation with aldehydes or with ethyl formate (Zaprutko 1999; Honda et al. 2000a; Govardhan et al. 1983; Yasue et al. 1973) to give products **5a**–**5c**, **6a**–**6c**. With phenylhydrazine oleanonic acid (**3**) as well as its methyl ester **4** form products with indole system: **7** and **8**, respectively (Finlay et al. 2002). This type of reaction leading to such compounds as **7** and **8** is known as Fisher indolization. Oleanonic acid (**3**) or its methyl ester (**4**) refluxed with 2,4-dinitrophenylhydrazine in acetic acid afford the appropriate phenylhydrazones (**9** and **10**, respectively). Heating of oleanonic acid (**3**) or methyl oleanonate (**4**) with hydroxylamine hydrochloride affords the appropriate oximes (**11** and **12**, respectively). Oxime function can be next subjected e.g. to the reaction of reduction, acylation, cyclization to oxazole ring, Beckmann rearrangement.

Similar transformations can be also performed for lupanes (e.g. betulin, betulinic acid, lupeol) and glycyrrhetinic acid.

According to our knowledge newly obtained *O*-acyloxyimino compounds are known only within lupanes and oleananes. Such type of compounds (with the acyloxyimino function) presents perspective pharmacological activities, such as anticancer, anti-HIV or anti-inflammatory.

## Acyloxyimino derivatives of lupanes

**Scheme 2:**
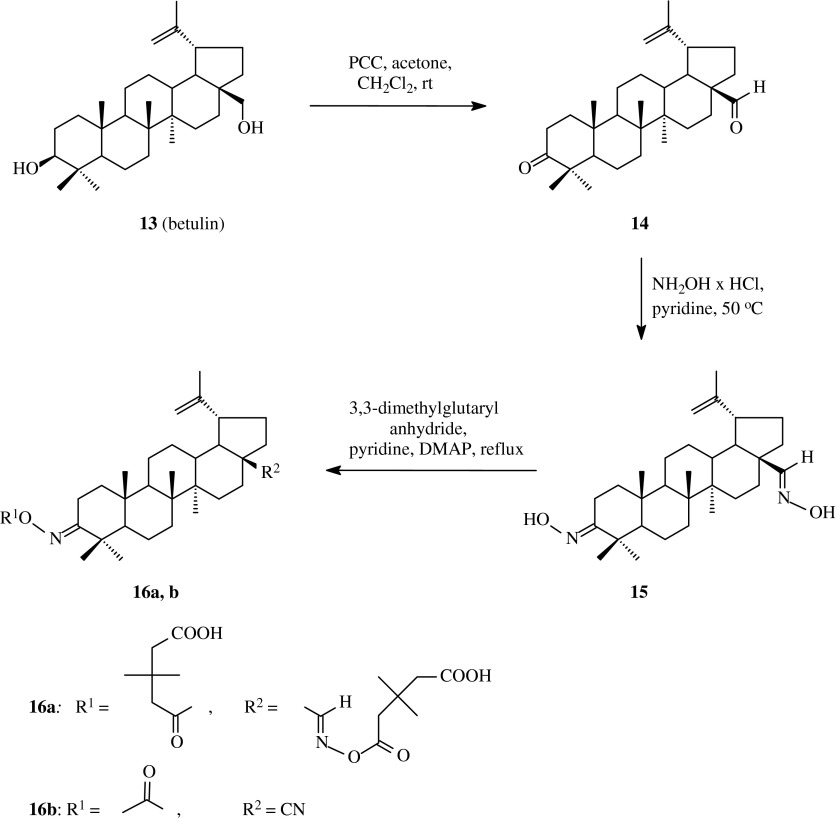
The synthesis of acylated oximes **16a**, **16b** derived from betulin (**13**)

In 1998 (Sun et al. 1998) the method of synthesis and the test of activity against HIV-1 virus of betulin derivatives were published. Betulin (**13**), one of the most often occured triterpene with lupane skeleton, was oxidizied with pyridinium chlorochromate (PCC) to betulonic aldehyde **14** (Scheme 2). The dicarbonyl compound **14** obtained with this method in the reaction with hydroxylamine hydrochloride formed dioxime **15**. Next the triterpene **15** was transformed into product **16a** with the appliance of 3,3-dimethylglutaryl anhydride and DMAP in refluxing pyridine, as presented in Table 1 (route 1).

**Table 1:** Reagents, their molar ratio, reaction conditions, specialized methods of purification and yields of *O*-acylderivatives of triterpenic oximes

Route no.	Reagents and their molar ratio	Reaction conditions	Specialized purification; yield	Ref.
1	Oxime **15**: 0.5 mmol DMAP: 0.5 mmol 3,3-Dimethylglutaryl anhydride: 8–10 mmol	The mixture of reagents in dried pyridine was heated at 95 °C overnight	Column chrom. (silicagel, CHCl_3_ and acetone = 13:1); Yield of **16a**: 59 %	Sun et al. (1998)
2	Oxime **20**, **21** or **22**: 1.0 mmol Carboxylic acid anhydride: 1.5 mmol Triethylamine: 0.4 ml	The mixture of reagents in dried benzene or diethyl eter was stirred for 4 h at room temp.	Column chrom. (Al_2_O_3_, CHCl_3_ and CH_3_OH = 10:1); Yields of **23a–23c**, **24a–24c**, **25a–25c** 64–77 %	Flekhter et al. (2004)
3	Oxime **20**: 1.0 mmol Carboxylic acid anhydride: 1.5 mmol DMAP: 0.2 mmol	The mixture of reagents in dried pyridine was refluxed for 12 h	Column chrom. (silicagel, CHCl_3_ and CH_3_OH = 99:1 or pure CHCl_3_); Yields of **23a** and **23e–23** **h**: 63–86 %	Genet et al. (2010)
4	Oxime **24**: 1.0 mmol 5-Phenyl-2,3-dihydrofuran-2,3-dione: 1.0 mmol	The mixture of reagents in dried toluene was refluxed for 30 min	No column chrom.; Yield of **26**: 70 %	Nekrasov and Obukhova (2005)
5	Oxime **11**: 1.0 mmol Adipoyl dichloride: 13.75 mmol	The mixture of reagents in dried THF was heated at 60 °C for 30 min	Twice column chrom. (1: RP-18, H_2_O and CH_3_OH, 2: silicagel, *n*-hexane and acetone = 9:1–7:3); Yield of **27a**: 36 %	Ma et al. (2000)
	Oxime **12**: 1.00 mmol Adipic acid: 9.25 mmol DCC: 2.50 mmol	The mixture of reagents in dried THF was stirred at room temp. for 3 h	Twice column chrom. (1: ODS, 2: silicagel, H_2_O and CH_3_OH = 1:1–100:0); Yield of **28a**: 97.3 %	
6	Oxime **11** or **12**: 0.03–0.06 mmol Adipoyl-AZT: 0.07 mmol DMAP: 0.06 mmol DCC: 0.08 mmol	The mixture of reagents in dried THF was stirred at room temp. overnight	Column chrom. (ODS), next: HPLC (H_2_O and CH_3_OH = 20:80–0:100; Yields: **27b**: 54.4 %, **28b**: 58.1 %	Ma et al. (2002)
	*O*-acyloxime **28a**: 0.02–0.05 mmol, DMAP: 0.03 mmol FK 3000: 0.02–0.05 mmol DCC:0.02–0.05 mmol	The mixture of reagents in dried methylene chloride was stirred at room temp. for 10 h	Twice column chrom. (1: RP-18, H_2_O and methanol = 3:7–0:1), 2: silicagel, *n*-hexane and acetone = 9:1–7:3); Yield of **28c**: 55.7 %	
7	*First step*: Oxime **30**: 1.0 mmol Sodium acetate: 1.12 mmol Na_2_PdCl_2_: 1.11 mmol *Second step*: Crude product of first step: 1.00 mmol DMAP: 0.05 mmol Trietylamine: 0.64 mmol *Third step*: Compound **31** obtained earlier, Pb(CH_3_COO)_4_: 4.59 mmol NaBH_4_: 180 mg 1 N NaOH	*First step*: the mixture of reagents in acetic acid was stirred at room temp. for 72 h; * Second step*: the mixture of reagents in acetic anhydride and CH_2_Cl_2_ was stirred at room temp. for 45 min * Third step*: crude product was dissolved in dried THF and pyridine and stirred at room temp. for 15 min, next it the was cooled to −78 °C and the solution of Pb(CH_3_COO)_4_ in CH_3_COOH was added. After that solution was stirred for 15.5 h at room temp. Next, a solution of NaBH_4_ in 1 N NaOH solution was added and stirred at room temp. for 10 min	*First step*: no column chrom.; Yields of **31**: no data; *Second step*: no purification; *Third step*: column chrom. (silicagel, *n*-hexane and ethyl acetate = 3:2; Yield of **32**: 56 % in comparison to oxime **30**	Honda et al. (2004)
8	Dioxime **39**: 1.0 mmol DMAP: 0.2 mmol Acetic anhydride: 20 mmol	The mixture of reagents in pyridine was stirred at room temp. for 24 h	Column chrom. (silicagel, CH_2_Cl_2_ and CH_3_OH = 70:1); Yield of **41**: 51 %	Hu et al. (2009)
9	Oxime **11**, **12**, **42** or **43**: 1.0 mmol Carboxylic acid: 1.2 mmol DCC: 1.5 mmol	The mixture of reagents in dried dioxane or THF was stirred at room temp. for 30–90 min	No column chrom.; Yields of acylated oximes **44a**–**44** **h**, **45a**–**45** **h**, **46a**–**46** **h** and **47a**–**47** **h**: 86–96 %	Bednarczyk-Cwynar et al. (2011)
10	Oxime **12**: 1.0 mmol Octanoic acid: 3.0 mmol DCC: 5.0 mmol	The mixture of reagents in dried THF was stirred at room temp. for 30 min	No column chrom.; Yield of **48**: about 93 %	Bednarczyk-Cwynar et al. (2012a)
11	Oxime **11** or **49**: 1.0 mmol Carboxylic acid: 1.2 mmol DCC: 1.4 mmol	The mixture of reagents in dried CH_2_Cl_2_ was refluxed for 8–14 h	Column chrom. (silicagel, petroleum ether with EtOAc); Yields of **50a–50j** and **51a–51o**: 70–93 %.	Zhao et al. (2013)
12	Oxime **53**: 1.0 mmol Carboxylic acid: 1.2 mmol DCC: 1.5 mmol	The mixture of reagents in dried dioxane was stirred at room temp. for 30 min	No column chrom.; Yield of **54a–e**: more than 90 %	Bednarczyk-Cwynar et al. (2012b)
13	Oxime **56**: 1.0 mmol Acetic or propionic anhydride: 1.5 mmol DMAP: catalytic	The mixture of reagents in pyridine was refluxed for 4–6 h	Column chrom. silicagel (chloroform with methanol = 200:1); Yields of **57a** and **57b**: about 70 %	Liu et al. (2007)

*DMAP* 4-(*N*,*N*-dimethyl)aminopyridine, *DCC* dicyclohexylcarbodiimide, *RP-18* reversed-phase with octadecyl carbon chain (C18)-bonded silica chromatography, *ODS* octadecylsilanyl chromatography, *AZT* azidothymidine, *HPLC* high-performance liquid chromatography

The newly obtained derivative **16a** as well as its mother dicarbonyl compound **15** were subjected to biological tests for anti-HIV activity in acutely infected H9 lymphocytes. For H9 lymphocytes the values of IC_50_ and EC_50_ for dioxime **15** were 5.47 and 1.07 μM, respectively (Table 2), and for diacylderivative **16a** were 15.4 and 4.57 μM, respectively.

**Table 2:** Pharmacological activity of acylated oximes of triterpenes and their mother compounds

Compound	Results	Activity	Literature
**13** (betulin)	IC_50_ = 43.7 μM	Antiviral (HIV acutely infected H9 lymphocytes)	Sun et al. (1998)
**15** (dioxime of **13**)	IC_50_ = 5.47 μM		
**16a** (di-*O*-acyl deriv. of **15**)	IC_50_ = 15.4 μM		
**13** (betulin)	EC_50_ = 23.0 μM	Antiviral (HIV acutely infected H9 lymphocytes)	
**15** (dioxime of **13**)	EC_50_ = 1.07 μM		
**16a** (di-*O*-acyl deriv. of **15**)	EC_50_ = 4.57 μM		
**13** (betulin)	VR = 7 %	Antiviral (SFV)	Pohjala et al. (2009)
**15** (dioxime of **13**)	VR = 121 %		
**16b** (di-*O*-acyl deriv. of **15**)	VR = 73 %		
**13** (betulin)	CV = 83 %		
**15** (dioxime of **13**)	CV = 80 %		
**16b** (di-*O*-acyl deriv. of **15**)	CV = 85 %		
**20** (oxime of BA)	EC_50_ = 2.17 μM	Antiviral (influenza A)	Flekhter et al. (2004)
**21** (oxime of MB)	EC_50_ > 25.84 μM		
**22** (oxime of 20-OAB)	EC_50_ = 150.54 μM		
**20** (oxime of BA)	MTC/EC_50_ = 392.16		
**21** (oxime of MB)	MTC/EC_50_ < 1		
**22** (oxime of 20-OAB)	MTC/EC_50_ = 2.83		
**20** (oxime of BA)	EC_50_ = 81.56 μM	Antiviral (HSV 1)	
**21** (oxime of MB)	EC_50_ = 31.32 μM		
**22** (oxime of 20-OAB)	EC_50_ = 160.07 μM		
**20** (oxime of BA)	MTC/EC_50_ = 1.30		
**21** (oxime of MB)	MTC/EC_50_ = 6.60		
**22** (oxime of 20-OAB)	MTC/EC_50_ = 2.66		
**20** (oxime of BA)	EC_50_ = 4.19 μM	Antiviral (ECHO 6)	
**21** (oxime of MB)	EC_50_ = 36.84 μM		
**22** (oxime of 20-OAB)	EC_50_ = 133.10 μM		
**20** (oxime of BA)	MTC/EC_50_ = 6.09		
**21** (oxime of MB)	MTC/EC_50_ = 11.22		
**22** (oxime of 20-OAB)	MTC/EC_50_ = 1.60		
BA	ED_50_ = 1.23 μg/ml	Cytotoxic (MOLT-4)	Mukherjee et al. (2004)
**20** (oxime of BA)	ED_50_ = 2.1 μg/ml		
**23a** (*O*-acyl deriv. of **20**)	ED_50_ = 2.2 μg/ml		
**23d** (*O*-acyl deriv. of **20**)	ED_50_ = 2.7 μg/ml		
BA	ED_50_ = 0.65 μg/ml	Cytotoxic (JurkatE6.1)	
**20** (oxime of BA)	ED_50_ = 1.8 μg/ml		
**23a** (*O*-acyl deriv. of **20**)	ED_50_ = 2.9 μg/ml		
**23d** (*O*-acyl deriv. of **20**)	ED_50_ = 4.9 μg/ml		
BA	ED_50_ = 0.98 μg/ml	Cytotoxic (CEM.CM3)	
**20** (oxime of BA)	ED_50_ = 2.6 μg/ml		
**23a** (*O*-acyl deriv. of **20**)	ED_50_ = 4.2 μg/ml		
**23d** (*O*-acyl deriv. of **20**)	ED_50_ = 1.5 μg/ml		
BA	ED_50_ = 0.84 μg/ml	Cytotoxic (BRISTOL8)	
**20** (oxime of BA)	ED_50_ = 1.6 μg/ml		
**23a** (*O*-acyl deriv. of **20**)	ED_50_ = 3.4 μg/ml		
**23d** (*O*-acyl deriv. of **20**)	ED_50_ = 3.3 μg/ml		
BA	ED_50_ = 0.69 μg/ml	Cytotoxic (U937)	
**20** (oxime of BA)	ED_50_ = 2.4 μg/ml		
**23a** (*O*-acyl deriv. of **20**)	ED_50_ = 5.5 μg/ml		
**23d** (*O*-acyl deriv. of **20**)	ED_50_ = 4.8 μg/ml		
BA	ED_50_ = 1.13 μg/ml	Cytotoxic (DU145)	
**20** (oxime of BA)	ED_50_ = 1.1 μg/ml		
**23a** (*O*-acyl deriv. of **20**)	ED_50_ = 2.9 μg/ml		
**23d** (*O*-acyl deriv. of **20**)	ED_50_ = 2.8 μg/ml		
BA	ED_50_ > 10 μg/ml	Cytotoxic (PA-1)	
**20** (oxime of BA)	ED_50_ = 0.7 μg/ml		
**23a** (*O*-acyl deriv. of **20**)	ED_50_ = 3.7 μg/ml		
**23d** (*O*-acyl deriv. of **20**)	ED_50_ = 1.8 μg/ml		
BA	ED_50_ > 10 μg/ml	Cytotoxic (A549)	
**20** (oxime of BA)	ED_50_ = 1.8 μg/ml		
**23a** (*O*-acyl deriv. of **20**)	ED_50_ > 10 μg/ml		
**23d** (*O*-acyl deriv. of **20**)	ED_50_ > 10 μg/ml		
BA	ED_50_ = 1.3 μg/ml	Cytotoxic (L132)	
**20** (oxime of BA)	ED_50_ = 1.5 μg/ml		
**23a** (*O*-acyl deriv. of **20**)	ED_50_ = 5.3 μg/ml		
**23d** (*O*-acyl deriv. of **20**)	ED_50_ > 10 μg/ml		
**20** (oxime of BA)	EC_50_ = 1.75 μM	Antidiabetic (TGR 5)	Genet et al. (2010)
**23a** (*O*-acyl deriv. of **20**)	EC_50_ = 5.00 μM		
**23e** (*O*-acyl deriv. of **20**)	EC_50_ = 3.17 μM		
**23f** (*O*-acyl deriv. of **20**)	EC_50_ = 7.88 μM		
**23g** (*O*-acyl deriv. of **20**)	EC_50_ = 7.73 μM		
**23h** (*O*-acyl deriv. of **20**)	EC_50_ = 3.65 μM		
**20** (oxime of BA)	efficacy = 132		
**23a** (*O*-acyl deriv. of **20**)	efficacy = 159		
**23e** (*O*-acyl deriv. of **20**)	efficacy = 20		
**23f** (*O*-acyl deriv. of **20**)	efficacy = 139		
**23g** (*O*-acyl deriv. of **20**)	efficacy = 126		
**23h** (*O*-acyl deriv. of **20**)	efficacy = 13		
**1** (OA)	IC_50_ = 8.0 μM	Antiviral (HIV-1)	Ma et al. (2000)
**11** (oxime of OA)	IC_50_ = 5.5 μM		
**12** (oxime of MO)	IC_50_ = 9.5. μM		
**27a** (*O*-acyl deriv. of **11**)	IC_50_ = 5.5. μM		
**28a** (*O*-acyl deriv. of **12**)	IC_50_ = 4.0 μM		
**1** (OA)	IC_50_ = 8.0 μM	Antiviral (HIV-1 PR)	Ma et al. (2002)
**11** (oxime of OA)	IC_50_ = 5.5 μM		
**12** (oxime of MO)	IC_50_ = 9.5 μM		
**27a** (*O*-acyl deriv. of **11**)	IC_50_ = 5.5 μM		
**27b** (*O*-acyl deriv. of **11**)	IC_50_ = 1.9 μM		
**28a** (*O*-acyl deriv. of **12**)	IC_50_ = 4.0 μM		
**28b** (*O*-acyl deriv. of **12**)	IC_50_ = 16 μM		
**28c** (*O*-acyl deriv. of **12**)	IC_50_ > 100 μM		
**1** (OA)	IC_100_ not effective	Antiviral (HIV-1 RT)	
**11** (oxime of OA)	IC_100_ not effective		
**12** (oxime of MO)	IC_100_ not effective		
**27a** (*O*-acyl deriv. of **11**)	IC_100_ not effective		
**27b** (*O*-acyl deriv. of **11**)	IC_100_ = 1.84 μM		
**28a** (*O*-acyl deriv. of **12**)	IC_100_ not effective		
**28b** (*O*-acyl deriv. of **12**)	IC_100_ = 4.53 μM		
**28c** (*O*-acyl deriv. of **12**)	IC_100_ not effective		
**1** (OA)	CC_0_ ≥ 110 μM	Antiviral (HIV-1 RT)	
**11** (oxime of OA)	CC_0_ = 266 μM		
**12** (oxime of MO)	CC_0_ = 51.8 μM		
**27a** (*O*-acyl deriv. of **11**)	CC_0_ = 209 μM		
**27b** (*O*-acyl deriv. of **11**)	CC_0_ = 29.6 μM		
**28a** (*O*-acyl deriv. of **12**)	CC_0_ = 40.9 μM		
**28b** (*O*-acyl deriv. of **12**)	CC_0_ = 145 μM		
**28c** (*O*-acyl deriv. of **12**)	CC_0_ = 31.0 μM		
**39** (dioxime of oleanonic	MGC = 1.20 g/100 cm^3^ (methylene chloride)	Gelation	Hu et al. (2009)
	MGC = 0.37 g/100 cm^3^ (chloroform)		
	MGC = 2.08 g/100 cm^3^ (carbon tetrachloride)		
	MGC = 0.10 g/100 cm^3^ (benzene)		
	MGC = 0.20 g/100 cm^3^ (toluene)		
**45d** (*O*-acyl deriv. of **12**)	GI_50_ = (1.55–12.80) × 10^−6^ the most sensitive cell line: HOP-92)	Anticancer (lung cancers)	Bednarczyk-Cwynar et al. (2011)
	GI_50_ = (1.66–34.80) × 10^−6^ (the most sensitive cell line: HCT-116)	Anticancer (colon cancers)	
	GI_50_ = (1.56–2.67) × 10^−6^ (the most sensitive cell line: HS-578T2)	Anticancer (brest cancers)	
	GI_50_ = (1.67–16.50) × 10^−6^ (the most sensitive cell line: OVCAR-3)	Anticancer (ovary cancers)	
	GI_50_ = (2.02–2.78) × 10^−6^ (the most sensitive cell line: HL-60 TB)	Anticancer (leukemias)	
	GI_50_ = (1.49–2.60) × 10^−6^ (the most sensitive cell line: UO-31)	Anticancer (kidney cancers)	
	GI_50_ = (1.47–11.50) × 10^−6^ (the most sensitive cell line: LOX IMVI)	Anticancer (melanomas)	
	GI_50_ = (1.76–10.40) × 10^−6^ (the most sensitive cell line: SF-539)	Anticancer (CNS cancers)	
	TGI = (4.04–22.90) × 10^−6^ (the most sensitive cell line: HOP-92)	Anticancer (lung cancers)	
	TGI = (3.02–12.60) × 10^−6^ (the most sensitive cell line: HCC-2998)	Anticancer (colon cancers)	
	TGI = (4.29–14.20) × 10^−6^ (the most sensitive cell line: MCF-7)	Anticancer (brest cancers)	
	TGI = (3.08–30.40) × 10^−6^ (the most sensitive cell line: OVCAR-3)	Anticancer (ovary cancers)	
	TGI = (5.36–8.16) × 10^−6^ (the most sensitive cell line: HL-60 TB)	Anticancer (leukemias)	
	TGI = (2.93–16.30) × 10^−6^ (the most sensitive cell line: UO-31)	Anticancer (kidney cancers)	
	TGI = (2.94–24.40) × 10^−6^ (the most sensitive cell line: LOX IMVI)	Anticancer (melanomas)	
	TGI = (3.37–22.10) × 10^−6^ (the most sensitive cell line: U251)	Anticancer (CNS cancers)	
	LC_50_ = (11.50–100.00) × 10^−6^ (the most sensitive cell line: HOP-92)	Anticancer (lung cancers)	
	LC_50_ = (6.14–38.80) × 10^−6^ (the most sensitive cell line: HCT-116)	Anticancer (colon cancers)	
	LC_50_ = (11.10–46.80) × 10^−6^ (the most sensitive cell line: MCF-7)	Anticancer (brest cancers)	
	LC_50_ = (5.70–60.20) × 10^−6^ (the most sensitive cell line: OVCAR-3.)	Anticancer (ovary cancers)	
	LC_50_ = (2.62–100.00) × 10^−6^ (the most sensitive cell line: HL-60 TB)	Anticancer (leukemias)	
	LC_50_ = (5.79–57.20) × 10^−6^ (the most sensitive cell line: UO-31)	Anticancer (kidney cancers)	
	LC_50_ = (5.89–51.50) × 10^−6^ (the most sensitive cell line: LOX IMVI)	Anticancer (melanomas)	
	LC_50_ = (6.39–54.90) × 10^−6^ (the most sensitive cell line: U251)	Anticancer (CNS cancers)	
**1** (OA)	MGIR = 22.4 %	Fungicidal (*Candida albicans* GlcN-6-P synthase inhibitor); conc. = 0.35 mM	Zhao et al. (2013)
**50a** (*O*-acyl deriv. of **11**)	MGIR = 29.4 %		
**50b** (*O*-acyl deriv. of **11**)	MGIR = 37.2 %		
**50c** (*O*-acyl deriv. of **11**)	MGIR = 40.8 %		
**50d** (*O*-acyl deriv. of **11**)	MGIR = 19.2 %		
**50e** (*O*-acyl deriv. of **11**)	MGIR = 30.8 %		
**50f** (*O*-acyl deriv. of **11**)	MGIR = 29.1 %		
**50** **g** (*O*-acyl deriv. of **11**)	MGIR = 22.9 %		
**50** **h** (*O*-acyl deriv. of **11**)	MGIR = 24.8 %		
**50i** (*O*-acyl deriv. of **11**)	MGIR = 21.0 %		
**50j** (*O*-acyl deriv. of **11**)	MGIR = 20.5 %		
**51a** (*O*-acyl deriv. of **49**)	MGIR = 8.7 %		
**51b** (*O*-acyl deriv. of **49**)	MGIR = 12.2 %		
**51c** (*O*-acyl deriv. of **49**)	MGIR = 19.8 %		
**51d** (*O*-acyl deriv. of **49**)	MGIR = 16.2 %		
**51e** (*O*-acyl deriv. of **49**)	MGIR = 20.2 %		
**51f** (*O*-acyl deriv. of **49**)	MGIR = 34.2 %		
**51** **g** (*O*-acyl deriv. of **49**)	MGIR = 28.2 %		
**51** **h** (*O*-acyl deriv. of **49**)	MGIR = 12.7 %		
**51i** (*O*-acyl deriv. of **49**)	MGIR = 19.3 %		
**51j** (*O*-acyl deriv. of **49**)	MGIR = 13.2 %		
**51** **k** (*O*-acyl deriv. of **49**)	MGIR = 16.4 %		
**51** **l** (*O*-acyl deriv. of **49**)	MGIR = 33.0 %		
**51** **m** (*O*-acyl deriv. of **49**)	MGIR = 33.1 %		
**51n** (*O*-acyl deriv. of **49**)	MGIR = 12.5 %		
**51o** (*O*-acyl deriv. of **49**)	MGIR = 13.8 %		
**1** (OA)	MGIR = 1.0–25.3 % (the most sensitive cell line: *Rhizoctonia solani* Kuhn)	Fungicidal; conc. = 50 μg/ml	
**50a** (*O*-acyl deriv. of **11**)	MGIR = 1.8–61.2 % (the most sensitive cell line: *Sclerotinia sclerotiorum* (Lib.) de Bary)		
**50b** (*O*-acyl deriv. of **11**)	MGIR = 8.4–61.2 % (the most sensitive cell line: *Sclerotinia sclerotiorum* (Lib.) de Bary)		
**50c** (*O*-acyl deriv. of **11**)	MGIR = 3.1–42.7 % (the most sensitive cell line: *Rhizoctonia solani* Kuhn)		
**50d** (*O*-acyl deriv. of **11**)	MGIR = 15.8–53.5 % (the most sensitive cell line: *Sclerotinia sclerotiorum* (Lib.) de Bary)		
**50e** (*O*-acyl deriv. of **11**)	MGIR = 6.5–73.0 % (the most sensitive cell line: *Rhizoctonia solani* Kuhn)		
**50f** (*O*-acyl deriv. of **11**)	MGIR = 4.0–33.5 % (the most sensitive cell line: *Rhizoctonia solani* Kuhn)		
**50** **g** (*O*-acyl deriv. of **11**)	MGIR = 6.1–67.6 % (the most sensitive cell line: *Phytophthora parasitica* Dast)		
**50** **h** (*O*-acyl deriv. of **11**)	MGIR = 2.0–27.8 % (the most sensitive cell line: *Sclerotinia sclerotiorum* (Lib.) de Bary)		
**50i** (*O*-acyl deriv. of **11**)	MGIR = 1.4–25.1 % (the most sensitive cell line: *Rice blast*)		
**50j** (*O*-acyl deriv. of **11**)	MGIR = 2.3–30.3 % (the most sensitive cell line: *Sclerotinia sclerotiorum* (Lib.) de Bary)		
**51a** (*O*-acyl deriv. of **49**)	MGIR = 9.4–79.2 % (the most sensitive cell line: *Rhizoctonia solani* Kuhn)		
**51b** (*O*-acyl deriv. of **49**)	MGIR = 12.0–71.1 % (the most sensitive cell line: *Sclerotinia sclerotiorum* (Lib.) de Bary)		
**51c** (*O*-acyl deriv. of **49**)	MGIR = 23.5–93.6 % (the most sensitive cell line: *Rhizoctonia solani* Kuhn)		
**51d** (*O*-acyl deriv. of **49**)	MGIR = 25.9–74.3 % (the most sensitive cell line: *Rice blast*)		
**51e** (*O*-acyl deriv. of **49**)	MGIR = 17.4–84.1 % (the most sensitive cell line: *Rhizoctonia solani* Kuhn)		
**51f** (*O*-acyl deriv. of **49**)	MGIR = 18.1–73.1 % (the most sensitive cell line: *Rhizoctonia solani* Kuhn)		
**51** **g** (*O*-acyl deriv. of **49**)	MGIR = 17.7–86.1 % (the most sensitive cell line: *Rhizoctonia solani* Kuhn)		
**51** **h** (*O*-acyl deriv. of **49**)	MGIR = 21.7–67.8 % (the most sensitive cell line: *Sclerotinia sclerotiorum* (Lib.) de Bary)		
**51i** (*O*-acyl deriv. of **49**)	MGIR = 25.3–74.4 % (the most sensitive cell line: *Sclerotinia sclerotiorum* (Lib.) de Bary)		
**51j** (*O*-acyl deriv. of **49**)	MGIR = 23.5–68.2 % (the most sensitive cell line: *Sclerotinia sclerotiorum* (Lib.) de Bary)		
**51** **k** (*O*-acyl deriv. of **49**)	MGIR = 21.4–64.5 % (the most sensitive cell line: *Sclerotinia sclerotiorum* (Lib.) de Bary)		
**51** **l** (*O*-acyl deriv. of **49**)	MGIR = 28.2–69.5 % (the most sensitive cell line: *Rhizoctonia solani* Kuhn)		
**51** **m** (*O*-acyl deriv. of **49**)	MGIR = 24.6–66.7 % (the most sensitive cell line: *Sclerotinia sclerotiorum* (Lib.) de Bary)		
**51n** (*O*-acyl deriv. of **49**)	MGIR = 30.8–77.6 % (the most sensitive cell line: *Rhizoctonia solani* Kuhn)		
**51o** (*O*-acyl deriv. of **49**)	MGIR = 28.7–85.5 % (the most sensitive cell line: *Rice blast*)		
**1** (OA)	IC_50_ = 14.93 μM	Anticancer (KB)	
**53** (oxime of OM)	IC_50_ = 2.06 μM		Bednarczyk-Cwynar et al. (2012b)
**54a** (*O*-acyl deriv. of **53**)	IC_50_ > 15.00 μM		
**54b** (*O*-acyl deriv. of **53**)	IC_50_ = 9.42 μM		
**54c** (*O*-acyl deriv. of **53**)	IC_50_ = 4.90 μM		
**54d** (*O*-acyl deriv. of **53**)	IC_50_ = 0.72 μM		
**54e** (*O*-acyl deriv. of **53**)	IC_50_ = 10.10 μM		
**1** (OA)	IC_50_ = 13.95 μM	Anticancer (MCF-7)	
**53** (oxime of OM)	IC_50_ = 11.27 μM		
**54a** (*O*-acyl deriv. of **53**)	IC_50_ > 15.00 μM		
**54b** (*O*-acyl deriv. of **53**)	IC_50_ = 7.26 μM		
**54c** (*O*-acyl deriv. of **53**)	IC_50_ = 3.76 μM		
**54d** (*O*-acyl deriv. of **53**)	IC_50_ = 2.13 μM		
**54e** (*O*-acyl deriv. of **53**)	IC_50_ = 9.28 μM		
**1** (OA)	IC_50_ = 11.82 μM	Anticancer (MCF-7)	
**53** (oxime of OM)	IC_50_ = 1.34 μM		
**54a** (*O*-acyl deriv. of **53**)	IC_50_ > 15.00 μM		
**54b** (*O*-acyl deriv. of **53**)	IC_50_ = 9.19 μM		
**54c** (*O*-acyl deriv. of **53**)	IC_50_ = 4.41 μM		
**54d** (*O*-acyl deriv. of **53**)	IC_50_ = 1.87 μM		
**54e** (*O*-acyl deriv. of **53**)	IC_50_ = 9.84 μM		
**55** (GA)	GI_50_ = 63.2 μM	Anticancer (HL-60)	Liu et al. (2007)
**56** (oxime of GA)	GI_50_ = 63.9 μM		
**57a** (*O*-acyl deriv. of **56**)	GI_50_ = 58.8 μM		
**57b** (*O*-acyl deriv. of **56**)	GI_50_ = 57.7 μM		
**55** (GA)	AC = 24.9 %		
**56** (oxime of GA)	AC = 24.5 %		
**57a** (*O*-acyl deriv. of **56**)	AC = 19.1 %		
**57b** (*O*-acyl deriv. of **56**)	AC = 27.7 %		

*BA* betulinic acid, *MB* methyl betulinate, *20-OAB* 20-oxoallobetulin, *OA* oleanolic acid, *MO* methyl oleanolate, *GA* glycyrrhetinic acid, *IC*_*50*_ 50 % inhibitory concentration, *EC*_*50*_ 50 % effective concentration, *VR* virus replication, *CV* cell viability, *MTC* maximum tolerable concentration, *ED*_*50*_ 50 % effective dose, *IC*_*100*_ 100 % inhibition concentration, *CC*_*0*_ minimum cytotoxic concentration, *MGC* minimum gelator concentration, *GI*_*50*_ 50 % growth inhibition, *TGI* total growth inhibitor, *LC*_*50*_ 50 % lethal concentration, *MGIR* mycelium growth inhibition rate, *AC* amount of apoptotic cells

The action of acetic anhydride at 120 °C for 2 h upon dioxime **15** (Pohjala et al. 2009) gave product **16b**, in which the oxime group at C-3 atom was also acetylated but in the same time the second oxime function, at C-28 position, was reduced to the nitrile one; this new compound **16b** was obtained with the yield of 34 %.

The primary screen of betulin, the above obtained derivatives **15** and **16b**, as well as another acyl derivatives of betulin against SFV (Semliki Forest Virus), combined with a counterscreen for Huh-7 cell viability, was run in order to determine the tentative inhibitory potential of each compound. The results of these tests were expressed as surviving fractions (remaining percentages of viral replication or cell viability) after exposure to each compound. Betulin (**13**) yielded 7 % virus SFV replication (Table 2) and 83 % cell Huh-7 viability; for dioxime **15** these values were 121 and 80 %, respectively. The introduction of acetoxyimino function instead of C-3 hydroxyl one and a nitrile moiety at C-28 led to 73 % virus replication and 85 % cell viability.

**Scheme 3:**
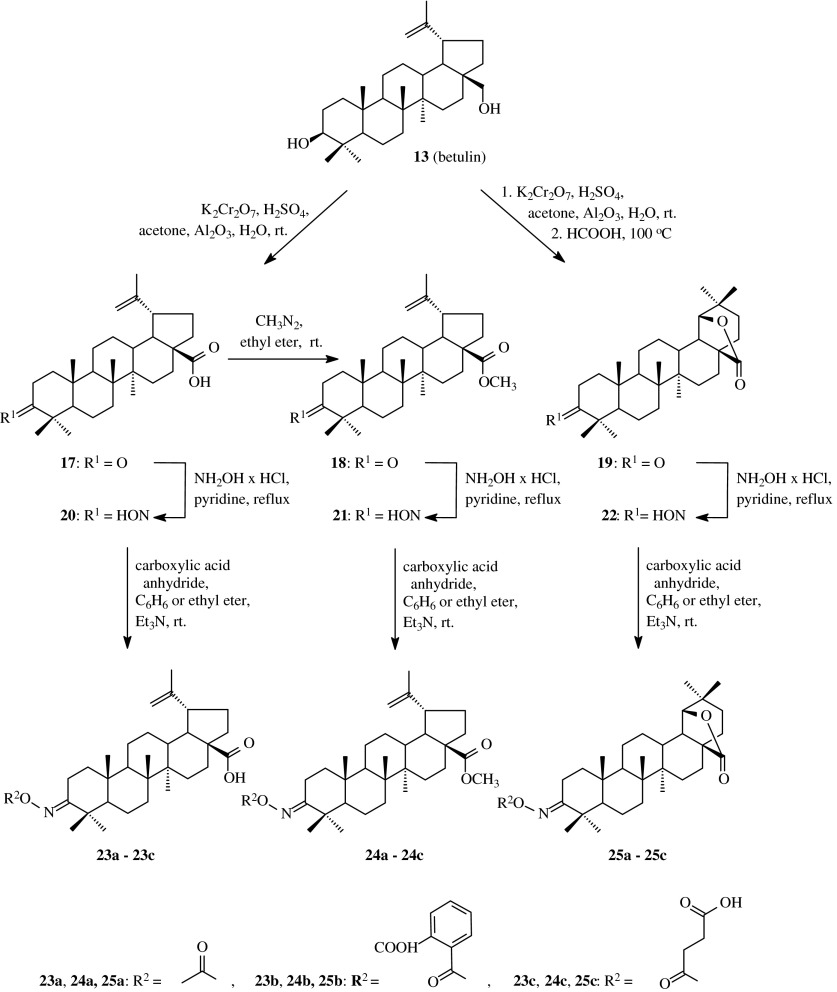
The synthesis of acylated oximes **23a–23c**, **24a–24c**, **25a–25c** derived from betulin (**13**)

The next information on acyloxyimino derivatives of lupane triterpenes was published in 2004 (Flekhter et al. 2004). In order to obtain such compounds, betulin (**13**) was transformed into betulonic acid (**17**), methyl betulonate (**18**) and 28-oxo-allobetulon (**19**) with the use of known methods (Scheme 3). The resulted 3-oxoderivatives **17**, **18** and **19** were subjected to the reaction with hydroxylamine hydrochloride in anhydrous pyridine which led to the obtaining of appropriate oximes (**20**, **21** and **22**) with high yields. The compounds **20**, **21** and **22** acylated in anhydrous benzene with carboxylic acid anhydrides (acetic, succinic or phthalic anhydride, Table 1, route 2) gave acylated oximes **23a**–**23c**, **24a**–**24c**, **25a**–**25c** with the yields from 64 % to 77 %.

The obtained oximes of betulin (**20**, **21** and **22**) were tested for their antiviral activity with the use of cell cultures inoculated with type 1 HSV virus, A/FPV/Rostock/34 (H7N1) influenza virus and ECHO-6 enterovirus. The antiviral effect was judged by reduction of the viral titer in the presence of test compounds in comparison to control sample and characterized by the effective concentration causing a 50 % inhibition of the virus growth (EC_50_) and by the ratio of a maximum tolerable drug concentration (MTC) to EC_50_. The MTC values were determined upon 72-h incubation of noninfected cell cultures. As the result showed, the most active compound turned to be the oxime of betulonic acid (**20**). The EC_50_ values with respect to viruses: H7N1, HSV-1 and ECHO-6 for this compound were, respectively: 2.17, 81.56 and 4.19 μM (Table 2) and the MTC values were, respectively: 392.16, 1.30 and 6.09. More active against HSV-1 virus was only the compound **21**, with methylated carboxyl function, for which EC_50_ value was 31.32 μM and MTC value was 6.60.

**Scheme 4:**
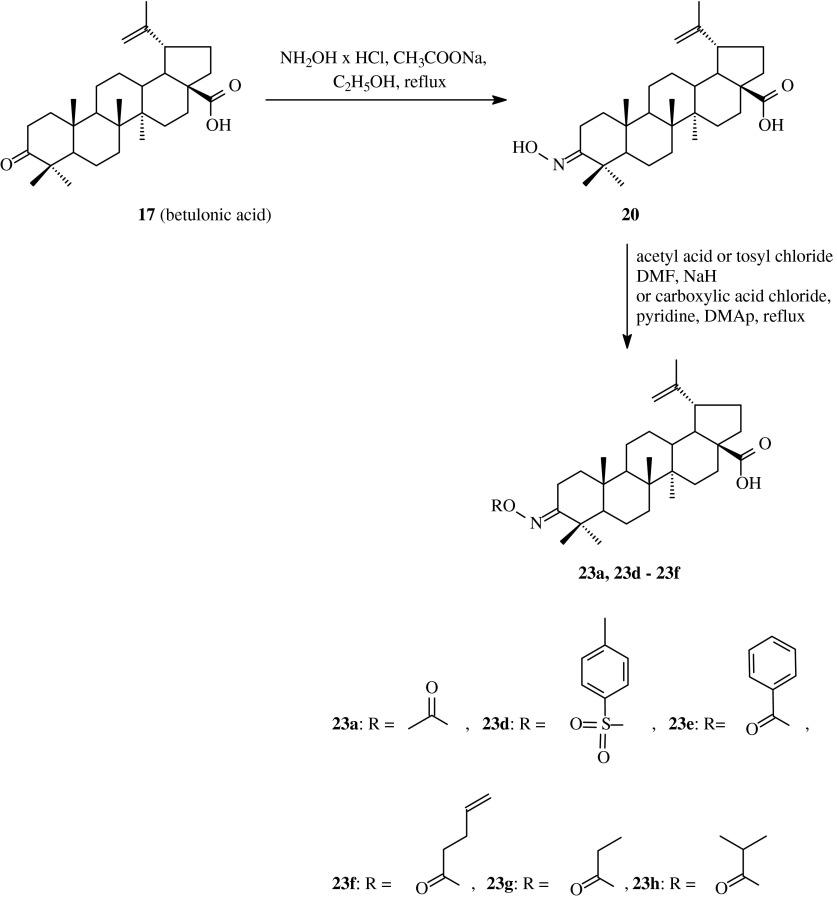
The synthesis of acylated oximes **23a** and **23d–23** **h** derived from betulonic acid (**17**)

The further study concerning acylated oximes of betulinates was published in the same year by Mukherjee and et al. (2004). Betulinic acid was converted into betulonic acid (**17**) upon the action of Jones reagent (Scheme 4) and then the reaction of the 3-oxo compound (**17**) with hydroxylamine hydrochloride in the presence of sodium acetate afforded the appropriate oxime **20**. The obtained hydroxyimino derivative **20** was subjected to the action of acetyl chloride or *p*-toluenesulfonyl chloride in DMF to obtain, respectively, compound **23a** known from the earlier presented publication and new product **23d**.

The obtained compounds **20**, **23a** and **23d** as well as another compounds obtained in this work, were subjected to biological tests to determine their cytotoxic activity against 9 lines of cancer cells: MOLT-4, Jurkat E6.1, CEM.CM3 (human lymphoblastic leukemias), BRISTOL8 (human B cell lymphoma), U937 (human histiocytic lymphoma), DU145 (human prostate cancer), PA-1 (human ovary cancer), A549 and L132 (human lung cancers). The ED_50_ values (50 % effective dose) for oxime **20** varied from 0.7 μg/ml for PA-1 cell line to 2.6 μg/ml for CEM.CM3 (Table 2), for acyloxyimino **23a** were from 2.2 μg/ml (MOLT-4) to more that 10 μg/ml (A549) and for derivative **23d** varied from 1.5 μg/ml (CEM.CM3) to >10 μg/ml (for A549 and L132 cell lines). The compounds **20** and **23d** were more active towards the tested cancer cell lines than betulinic acid only for PA-1 cell line (ED_50_ for betulinic acid for PA-1 was >10 μg/ml); ED_50_ values for derivatives **20** and **23d** and for PA-1 cell line were, respectively, 3.7 and 1.8 μg/ml.

Genet et al. (2010) synthesized further acyl derivatives of betulonic acid oxime **20** upon the action of carboxylic acid anhydrides in refluxed pyridine (Scheme 4, **23a** and **23e**–**23h**; Table 1, route 3). The yields of the synthesized acylated oximes of betulonic acid **23a** and **23e**–**23h** were from 63 to 86 %.

The obtained compounds were evaluated for their TGR5 agonist potency expressed as EC_50_ and as an efficacy. TGR5 is a recently identified G-protein coupled receptor which attracts scientific attention in the field of glucose metabolism. This receptor agonist could be used to treat type 2 diabetes because its activation is capable to increase the mitochondrial activity, which deficiency is the feature of the early stage of the diabetes. The EC_50_ value for compounds **23a** and **23e**–**23h** varied from 3.17 to 7.88 μM (Table 2) and the efficacy from 13 to 159 %, in comparison to mother betulinic acid (**17**), with an EC_50_ of 1.04 μM and an efficacy of 83 %.

**Scheme 5:**
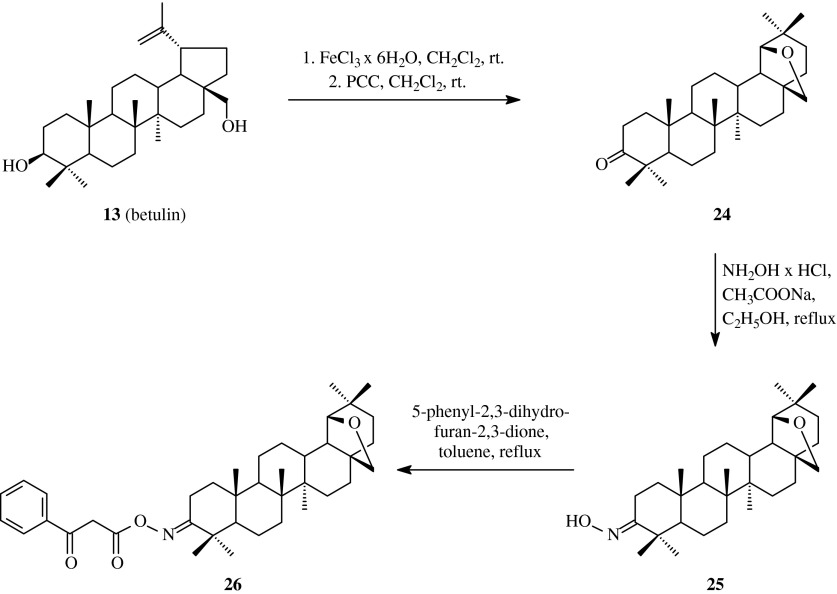
The synthesis of acylated oxime **26** derived from betulin (**13**)

As Nekrasov et al. reported (2005), allobetulon oxime **25** obtained from betulin **13** and refluxed for 30 min in anhydrous toluene with equimolar amount of 5-phenyl-2,3-dihydrofuran-2,3-dione gave unexpected product **26** (Scheme 5).

Usually, primary and secondary alcohols open up the ring of 5-aryl-2,3-dihydrofuran-2,3-diones at room temperature, to form the corresponding aroylpyruvic acid esters. To the contrary, allobetulone oxime (**25**) subjected to the action of 5-phenyl-2,3-dihydrofuran-2,3-dione after decarboxylation gave *O*-benzoylacetic acid derivative **26** (Table 1, route 4).

## Acyloxyimino derivatives of oleananes

There are also known acylated oximes derived from triterpenes of oleanane skeleton-oleanolates, maslinates and glycyrrhetinates.

## Acyloxyimino derivatives of oleanolates and maslinates

**Scheme 6:**
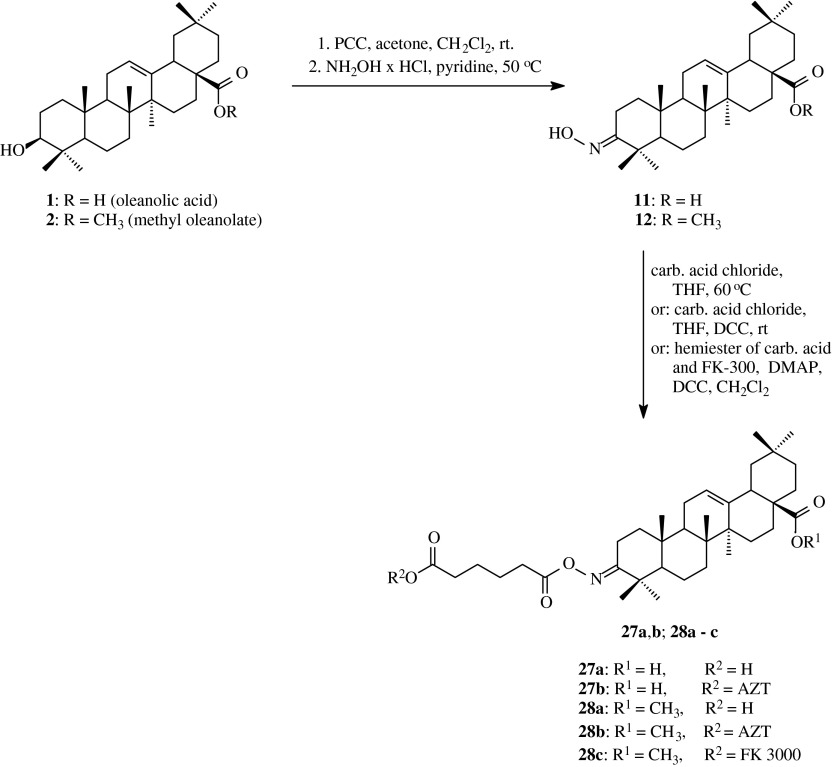
The synthesis of acylated oximes **27a**,**b** and **28a–28c** derived from oleanolic acid (**1**)

The first derivatives of this type were synthesized and published in 2000 (Ma et al. 2000). Oleanolic acid (**1**) or its methyl ester (**2**) were oxidizied with pyridinium chlorochromate and the resulted products (**3** and **4**) were subjected to the action of hydroxylamine hydrochloride in pyridine (Scheme 6). Next, oxime **11** and **12** were transformed into their *O*-acyl derivatives **27a** and **28a**, respectively, upon the action of adipoyl dichloride in heated THF or adipic acid and DCC in THF at room temperature (Table 1, route 5). Compounds **27a** and **28a** were obtained with the yields, accordingly, 36 % and almost 98 %.

The obtained derivatives of oleanolic acid (**27a** and **28a**) were tested towards their activity as inhibitors of HIV-1 dimerization protease (HIV-1 PR). It was stated that the introduction of acyl function with six-membered chain into hydroxyimino group intensifies the inhibitory effect of the tested compound against the above mentioned enzyme (Table 2). The IC_50_ values for oleanolic acid was 8.0 μM, and for acylated oximes **27a** and **28a**, were 5.5 and 4.0 μM, respectively.

Kimura et al. (1999) decided to obtain new compounds in which triterpenic molecule with HIV-1 PR inhibitory activity would be be joined with non-terpenic molecule with HIV-1 TR inhibitory activity, such as azidothymidine (AZT). The product of this type should show much better permeability into cells, moreover, within the cell the molecule of such conjugate would break down into two elements of different types of activity against HIV-1 so would be excellent antiviral agent.

Ma et al. (2002) acylated the oximes: **11** and **12** also with hemiester of adipic acid and AZT or FK-3000 (Scheme 6). Both, AZT and FK-3000, are well known medicines applied in HIV-1 therapy. FK-3000 is an alkaloid of morphine type, it shows the activity against *Herpex simplex* virus and inhibits the synthesis of viral DNA. Moreover, this compound acts as an inhibitor of reverse transcriptase of HIV-1 virus with IC_100_ value of 7.8 μg/ml (IC_100_—the minimum concentration for complete inhibition of HIV-1 induced CPE in MT-4 cells by microscopic observation) and CC_0_ value: 15.6 μg/ml (CC_0_—the minimum concentration for appearance of MT-4 cell toxicity by microscopic observation), but at the same time it is not active towards HIV-1 dimerization protease (Ma et al. 2002).

Ma et al. (2002) obtained the oximes **11** and **12** with the appliance of the earlier described method (Ma et al. 2000). The reaction of oxime acylation was performed as presented in Table 1, route 6.

The resulted triterpenic derivatives were tested towards their activity as HIV-1 PR inhibitors and HIV-1 RT inhibitors. The inhibitory activity against HIV-1 dimerization protease was evaluated by HPLC analysis of the cleavage products of a synthetic substrate. The IC_50_ value for AZT was above 100 μM (Table 2), for oximes acylated with adipic acid (**11** and **12**) were 5.5 and 4.0 μM, respectively, for compounds with AZT system (**27a** and **28a**) were 1.9 and 16.0 μM, respectively, and for acylated oxime with FK-3000 system (**28c**) the IC_50_ value also exceeded 100 μM; it means that this compound in fact was inactive as HIV-1 PR inhibitor.

The inhibitory activity against HIV-1 reverse transcriptase of the obtained conjugated compounds was tested with the use of MT-4 cells. The results were shown as IC_100_ and CC_0_ values.

The derivatives **27a** and **28a** turned to be practically inactive, with CC_0_ values 209.0 and 40.9 μM, respectively (Table 2). The introduction of azidothymidine system into the molecule of triterpene significantly increased the activity of compounds: IC_100_ values for such compounds (**27b** and **28b**) were: 1.84 and 4.53 μM, respectively, the CC_0_ values were 145.0 and 120.0 μM, respectively. The triterpenic derivative with FK-3000 system (**28c**) was inactive, with CC_0_ value 31.0 μM.

The insignificant inhibitory activity of product **28c**, both, against reverse transcriptase of HIV-1 and dimerization protease of HIV-1 (Table 2) can be caused by limited permeation of triterpene into cell and/or by high stability of amidic bound within this compound which makes impossible the cleavage of the molecule into two elements with different ways of activity against HIV-1 virus.

**Scheme 7:**
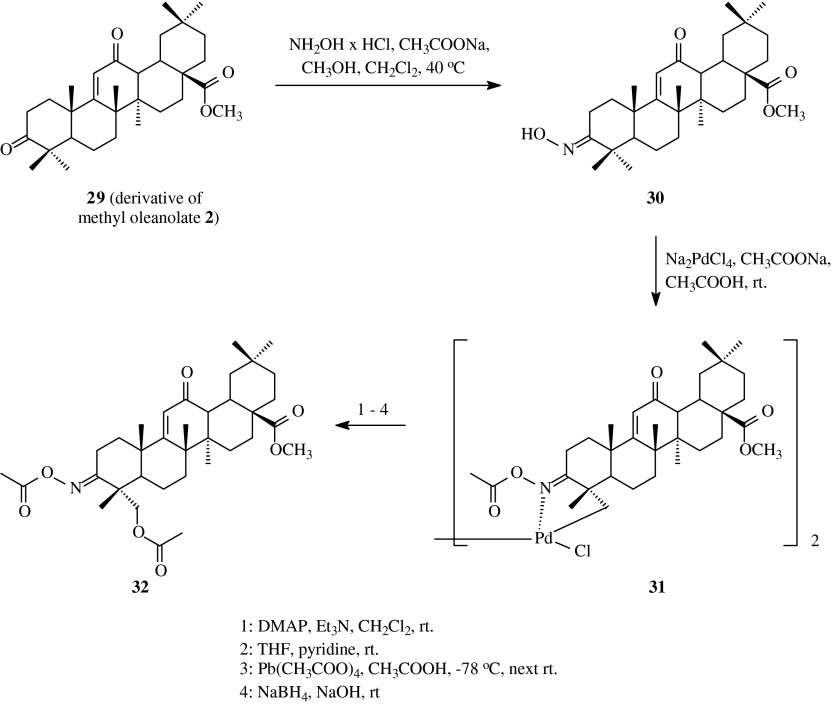
The synthesis of acylated oximes **31** and **32** derived from oleanolic acid (**1**)

The next acylated oximes of oleanolic acid were obtained by Honda et al. (2004) (Scheme 7). The diketone **29** was obtained as a result of six-step set of reactions, starting from methyl oleanolate **2** (Honda et al. 2000b). Compound **29** was subjected to the reaction with hydroxylamine hydrochloride in refluxed methanol with methylene chloride. The product of this reaction was compound **30** with hydroxyimino group only at C-3 position.

In the next steps, monooxime **30** was transformed into complex **31** and after that into diacetoxy derivative **32**, as it is presented in Table 1, route 7. Crude final product **32** was obtained with the yield of 56 % in comparison to oxime **30**.

**Scheme 8:**
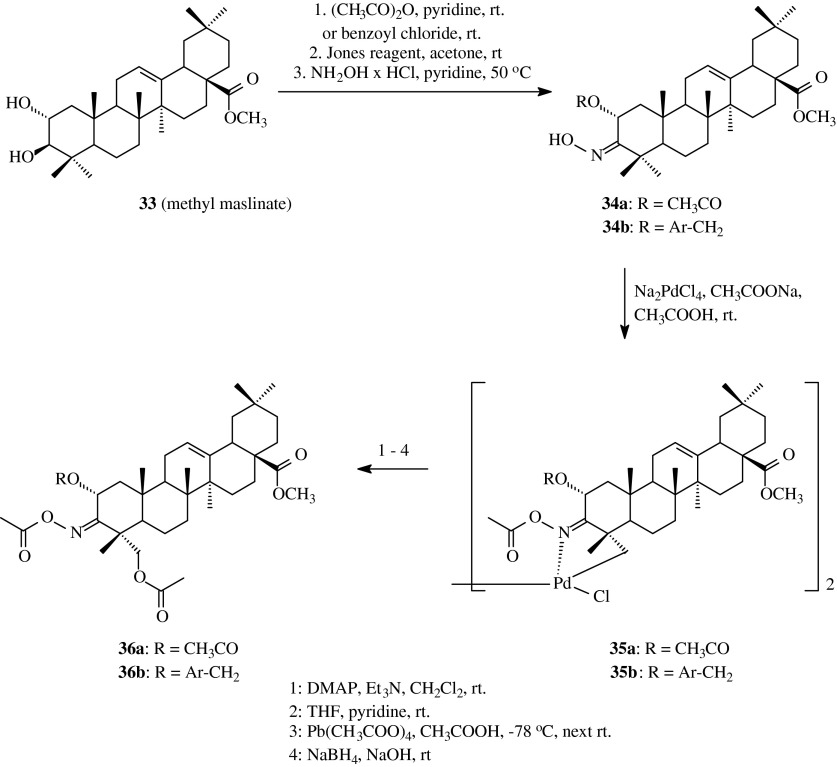
The synthesis of acylated oximes **35a**,**b** and **36a**,**b** derived from methyl maslinate (**33**)

The similar acyloxyimino derivative obtained via palladium complex as **31** was synthesized also for maslinic acid methyl ester (**33**) (García-Granados et al. 2007), which differs from oleanolic acid methyl ester (**2**) only in the presence of an additional α-hydroxyl group at C-2 position (Scheme 8). Starting from methyl maslinate (**33**), after blocking of C-2 hydroxyl group, the appropriate oximes (**34a**, **34b**) were obtained. Next, the above compounds (**34a** and **34b**) were transformed into acetyloxyimino derivatives (**36a** and **36b**, respectively) in the same conditions and by means of using of the same reagents as for methyl oleanolate (**2**) (Table 1, route 7). The above compounds, **36a** and **36b**, were received with the yields of 44 and 35 %, respectively.

Hu et al. (2009) obtained derivatives of oleanolic acid with two oxime groups which were then acylated.

**Scheme 9:**
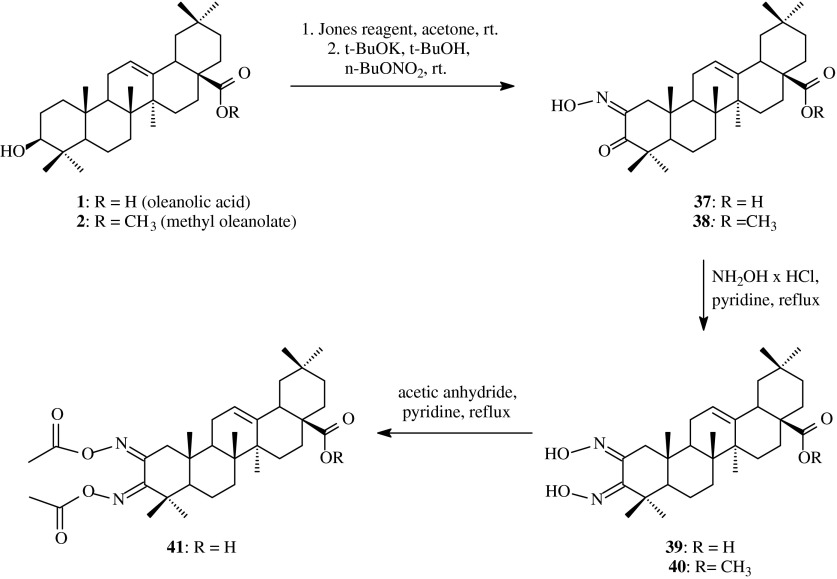
The synthesis of acylated oxime **41** derived from oleanolic acid (**1**)

Oleanolic acid (**1**) or its methyl ester (**2**) were oxidizied with Jones reagent in acetone to the appropriate ketones **3** and **4**, respectively. Next the oxoderivatives **3** and **4** were subjected to the action of *n*-butyl nitrite and as a result *α*-ketooximes **37** and **38**, respectively, were obtained (Scheme 9). These compounds were next refluxed in pyridine with hydroxylamine hydrochloride and yielded *α*-dioximes: **39** and **40**, respectively. The compound **39** reacted with acetic anhydride at room temperature in pyridine in the presence of DMAP (Table 1, route 8) to afford diacetyloxime **41** with the yield of 51 %.

The last compound **41** as well as dioxime **39** were tested to estimate their possibility to form organogel (Hu et al. 2009). This type of compounds are faced with many interest nowadays because of their interesting possibilities of application.

The gel formation ability was determined by a “stable to inversion of the test-tube” method. Specific amount of the tested compound was added into 15 different organic solvents at room temperature and these mixtures were heated to form transparent liquid, then cooled to room temperature to observe whether or not the immobile gel was formed in the inverted test-tube.

As it was shown on the basis of the obtained results, complete gel formation occurred in benzene, toluene and simple chlorinated solvents, such as methylene chloride, chloroform or carbon tetrachloride and the best solvent for the gelation process turned out to be benzene, in which gelation of dioxime **39** at a concentration as low as 0.10 g/100 cm^3^ occurs within 5 min (Table 2). Another organogels, obtained with the use of other organic solvents, were formed at concentrations from 0.2 g/100 cm^3^ to 2.08 g/100 cm^3^. For dioxime **41** gelation was not observed. It suggested that the presence of free hydroxyimino and carboxyl groups is favourable for gelation process.

When the stable gel formed in benzene was treated with HCl or gaseous NH_3_, it became unstable and eventually transformed into a suspension. This effect was caused by the deprotonisation or protonisation of the hydroxyimino and carboxyl groups within the molecule of dioxime **39** and the salts were formed. All these results confirmed the earlier thesis that the free hydroxyimino and carboxyl groups were significant to the gelation and the intermolecular hydrogen bonding played a crucial role in the formation of stable gels.

**Scheme 10:**
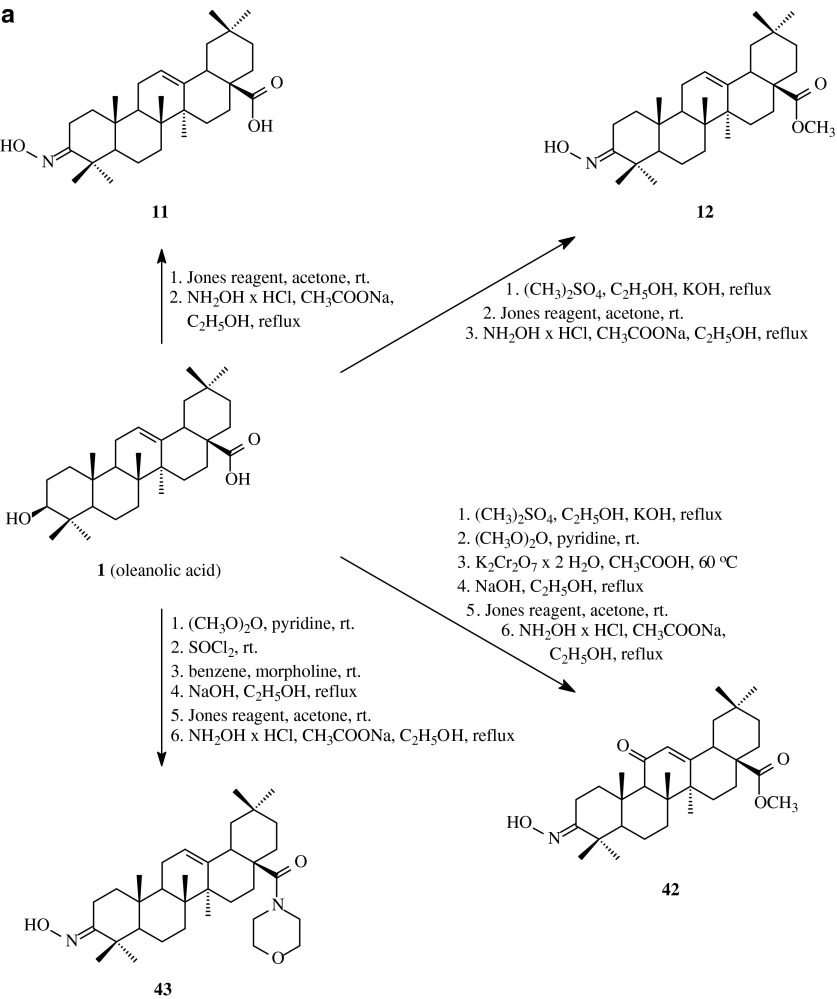

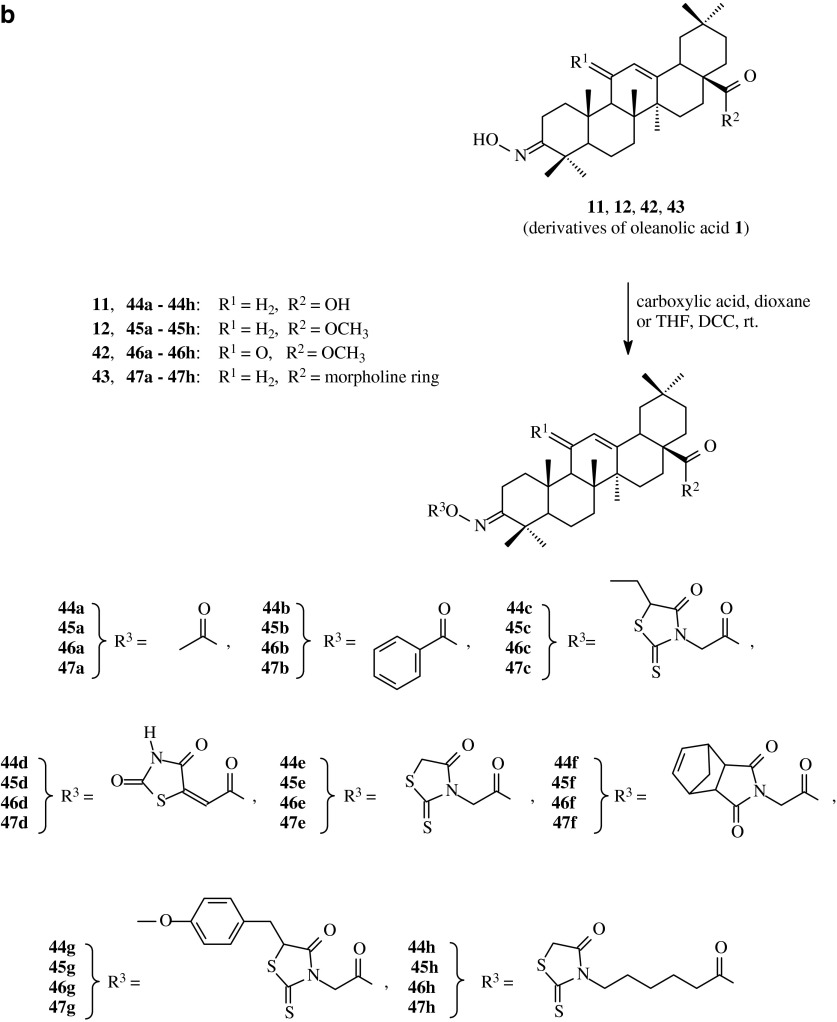
**a** The synthesis of oximes **11**, **12**, **42** and **43** derived from oleanolic acid (**1**). **b** The synthesis of acylated oximes **44a–44h**, **45a–45h**, **46a–46h** and **47a–47h** derived from oleanolic acid (**1**)

Acyl derivatives of oleanolic acid oximes can also be effective anticancer agents. With a view to obtain compounds with such activity, Bednarczyk-Cwynar et al. (2011) transformed oleanolic acid (**1**) and its methyl ester (**2**) into appropriate oximes (**11**, **12**, **42** and **43**, Scheme 10a), also with C-11 oxo group, with the use of modified methods from literature (Ma et al. 2000; Yasue et al. 1974).

The received oximes **11**, **12**, **42** and **43** (Scheme 10a) were subjected to acylation with the appliance of carboxylic acids in dried dioxane or THF in the presence DCC at room temperature (Table 1, route 9). Pure acylated oximes of oleanolic acid (**44a**–**44h**, **45a**–**45h**, **46a**–**46h** and **47a**–**47h**) were obtained with the yields of 86–96 %.

The oximes of oleanolic acid **11**, **12**, **42** and **43** as well as their derivatives obtained by acylation of the above oximes with heterocyclic carboxylic acids (**44c**–**h**, **45c**–**h**, **46c**–**h**, **47c**–**h**) were subjected to the biological tests in order to estimate their cytotoxic acitivity against selected cancer cell lines. The tests were conducted with the use of doxorubicin eluting beads (DEB) and the procedure according to NCI methods (Monks et al. 1991; Boyd and Paull 1995), in which the cancer cell lines of different type of lung, colon, breast, ovary, kidney, prostate, CNS as well as leukemia and melanoma, provided a research material. Anticancer activity of the tested compounds was determined with the use of spectrophotometric method based on sulphorodamine B as an agent linked selectively to proliferating proteins of the tested cells. The results were presented as a percentage of increase inhibition of cancer cells caused by the action of the tested cytostatic compounds.

The tested acyloxyimino triterpenes turned to be moderately active towards most of the used cell lines, the decrease of the amount of surviving cancer cells was in some cases higher than 50 %. On the basis of the obtained results it can be stated that the anticancer activity of the tested triterpenes is dependent on the structure of carboxylic acid connected to oxime moiety. The most active compounds were **45d**, **45e**, **45g** and **45h**, all of them with esterified carboxyl group within the triterpene moiety and with thiazolidine ring within acyl group. The highest level of anticancer activity exhibited the derivative **45d**, so taking under consideration the structure of the obtained products and their anticancer activity, it can be stated that the advantageous element of the structure, influencing on the level of anticancer activity, can be N–H group within thiazolidine ring. The decrease of the amount of cancer cells exceeded 50 % for most types of lungs and breast cancer lines as well as for almost all types of leukemia.

For the derivative **45d**, with the highest level of anticancer activity, the additional biological tests were performed for the concentrations from 10^−4^ to 10^−8^ M. On the basis of the obtained data the most characteristic parameters: GI_50_, TGI and LC_50_ were estimated.

The GI_50_ values for the most sensitive cell lines ranged from 1.47 × 10^−6^ (LOX IMVI cell line) to 2.02 × 10^−6^ for HL-60 TB (Table 2); TGI values were between 2.93 × 10^−6^ (UO-31 cell line) and 5.36 × 10^−6^ (HL-60 TB) and the LC_50_ values were found from 2.62 (HL-60 TB) to 11.5 (HOP-92). The obtained results of the cytotoxicity of the oleanolic acid acylated oximes expressed in a form of the above parameters, pointed at potent antileukemic activity of the triterpenic oximes acylated with some carboxylic acids containing thiazolidone moiety. In molecules of the most potent antileukemic products thiazolidone skeleton was joined with carboxyl function via short carbon chain.

**Scheme 11:**
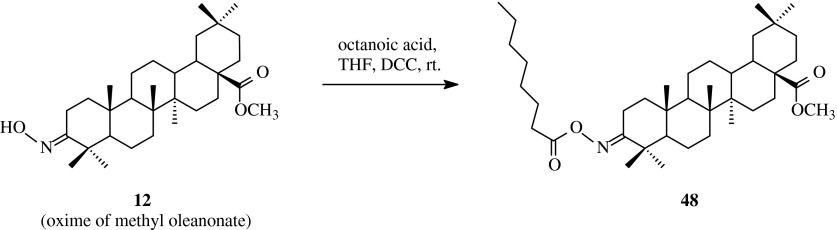
The synthesis of acylated oxime **48** derived from oleanolic acid (**1**)

As the literature data proved (Bednarczyk-Cwynar et al. 2012a) the methyl oleanolate 3-oxime acylated with fatty acid can be active analgesic and anti-inflammatory agent. In order to obtaine this type of compound oleanolic acid (**1**) was transformed into its methyl ester oxime (**12**) in three-step set of reactions (Scheme 11). The obtained hydroxyimino compound (**12**) was acylated with octanoic acid in the presence of DCC in THF (Table 1, route 10). The product **48** was received with the yield of about 93 %.

The new compound **48** was subjected to acute toxity, locomotor activity, analgesis activity and anti-inflammatory tests.

For toxicity studies, the group of mice were administered triterpene **48** (p.o.) in the dose of 2.0 g/kg. It was found that the acute administration of **48** did not cause any changes in skin and mucous appearance, cardiovascular, respiratory and central nervous system function and mortality during the next 14 days proving non toxic effects upon mice. In locomotor activity tests, horizontal and vertical locomotor activities of mice were evaluated using an activity meter which recorded spontaneous activity of animals in the time of 30 and 60 min after triterpene **48** administration in the dose of 3.0, 30.0, 100.0 and 300.0 mg/kg intragastrically (p.o.). It was found that compound **48** did not affect horizontal activity of mice in doses of 3.0, 30.0 and 100.0 mg/kg in both periods, but after administration of 300.0 mg/kg m.c. an increase in activity was observed. Moreover, similar effects in vertical activity were observed.

In analgesic activity test pain reflexes of mice in response to a thermal stimulus were measured in hot plate test after the same way of application and the same doses of triterpene **48** in the time of 30, 60, 90 and 120 min following the application. For comparative purposes, morphine in a dose of 5.0 mg/kg (s.c.) was administered to mice. It was observed that the compound **48** showed analgesic activity in the dose of 300.0 mg/kg and 60 min after administration. The combined treatment of triterpene **48** and morphine showed stronger analgesic activity in the doses of 30.0 and 300.0 mg/kg in comparison to morphine effect after 60 min.

In the next test, skin inflammation was induced in the right hindpaw of rats by the topical application of 2 mg/paw of carrageenan dissolved in 0.2 ml of 0.9 % saline solution. To the control, the rear left paw of the rats received the same volume of 0.9 % saline solution. Single doses of the compound **48** in range of 0.3, 3.0, 30.0 and 300.0 mg/kg were given intragastrically (p.o.) 30 min after carrageenan injection. For comparison, one group of rats was treated with the acute diclofenac (50 mg/kg, i.p.) injected 60 min after carrageenan administration. The interaction between methyl 3-octanoyloxyiminoolean-12-en-28-oate (**48**) (30 mg/kg, p.o.) and diclofenac was also tested. The rate of oedema of the two paws was measured at 1.5, 3.0, 6.0, and 10.0 h after carrageenan injection using a plethysmometer. The triterpene **48** caused a significant antioedemic effect in all doses after 1.5 and 3 h after carrageenan application in comparison to the control. The effect was also observed in three smallest doses after 6 h and in the dose of 3.0 mg/kg after 10 h from carrageenan application.

**Scheme 12:**
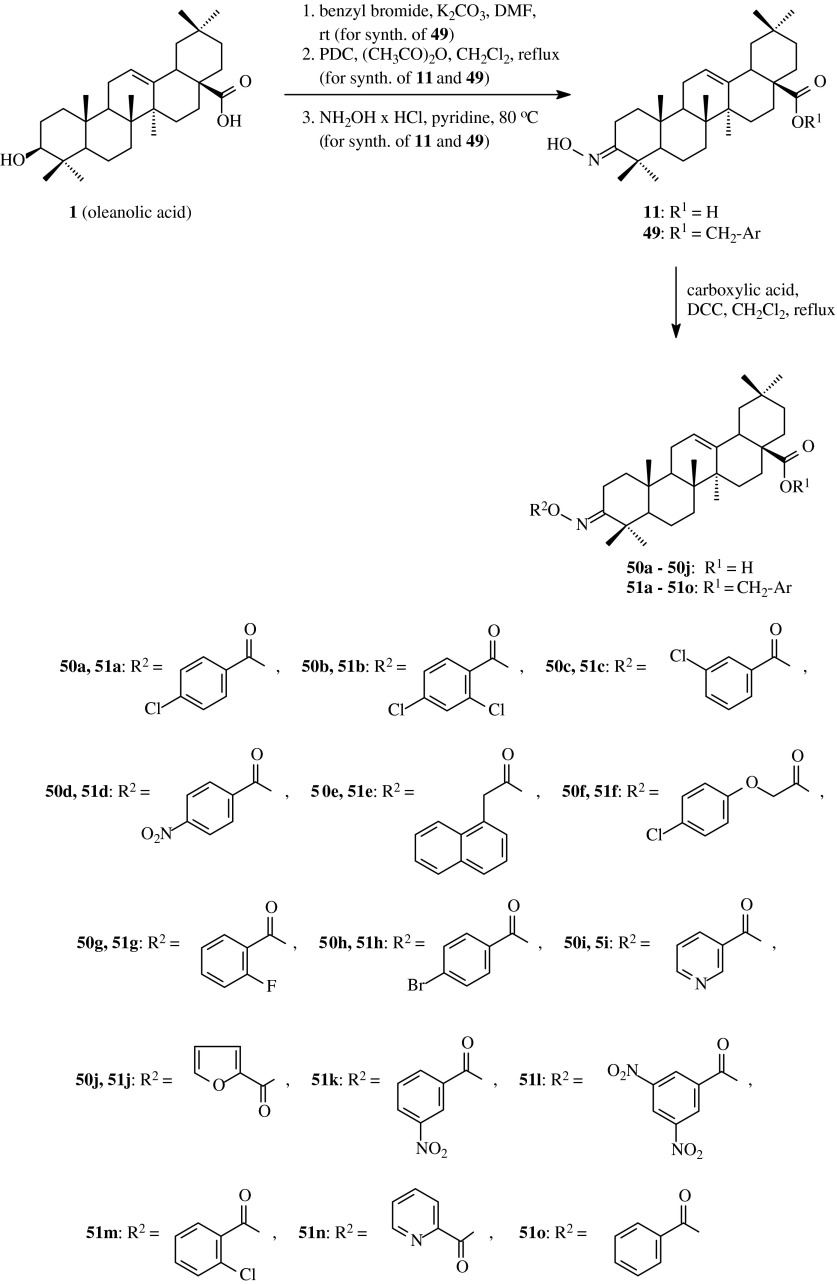
The synthesis of acylated oximes **50a–50j** and **51a–51o** derived from oleanolic acid (**1**)

Zhao et al. (2013) proved moderate antifungal activity of acylated oximes of oleanolic acid. In order to obtain these compounds, oleanolic acid (**1**) was transformed into its methyl or benzyl esters and next into the appropriate oximes **11** and **49**, respectively (Scheme 12). Next, hydroxyimino derivatives **11** and **49** were subjected to the action of carboxylic acids in refluxed methylene chloride in the presence of DCC to give the desired products **50a**–**50j** and **51a**–**51o** with the yields of 70–93 % (Table 1, route 11).

Zhao et al. (2013) presented that preliminary studies based on Elson–Morgan method (Roseman and Dafner 1956) proved some inhibitory activity of glucosamine-6-phosphate synthase (GlmS) of many compounds among the triterpenic derivatives **50a**–**50j** and **51a**–**51o**. The GlmS is the enzyme that plays a key role in the biosynthesis of the bacterial and fungal saccharides that are crucial elements of cell wall. Inhibitory activity of all the synthesized compounds was tested towards *Candida albicans* GlcN-6-P synthase. The Elson-Morgan method was based on colorimetry where the absorption value of the solution was measured at 585 nm, and then the concentration was counted on the basis of calibration curve.

Among the compounds **50a**–**50j** and **51a**–**51o** tested by Zhao et al. (2013) only the derivatives **50b**, **50c**, **51f**, **51l** and **51m** turned out to be more active against glucosamine-6-phosphate synthase than oleanolic acid (**1**) and exhibited the inhibition rate at 0.35 mM, respectively 37.2, 40.8, 34.2, 330 and 33.1 % (Table 2) in comparison to 22.4 % for mother oleanolic acid (**1**) applied in the same concentration.

The fungicidal activity of the obtained oleanolic acid acylated oximes **50a**–**50j** and **51a**–**51o** Zhao et al. evaluated against *Sclerotinia sclerotiorum (Lib.) de Bary*, *Phytophthora parasitica Dast*, *Botrytis cinerea Pers*, *Rhizoctonia solani Kuhn*, *Rice blast* and *Fusarium wilt* using the mycelium growth rate test (Zhao et al., 2013). The diameter of the mycelia was measured and the inhibition rate was calculated.

The inhibition rate for *S. sclerotiorum* ranged from 24.8 % (for **50i**) to 74.4 % (for **51i**), for *P. parasitica* was from 1.8 % (**50a**) to 67.6 % (**50g**) (Table 2), for *B. cinerea* ranged from 1.4 % (**50i**) to 69.1 % (**51i**), for *R. solani* from 5.5 % (**50j**) to 93.6 % (**51c**), for *Rice blast* waried from 21.0 % (**50e**, **50f**) to 85.5 % (**51o**) and for *Fusarium wilt* ranged from 5.1 % (**50j**) to 36.7 % (**51c**). In the same conditions oleanolic acid (**1**) exhibited the inhibition rate for this 6 fungi from 1.0 % (for *B. cinerea*) to 25.3 % (for *R. solani*).

**Scheme 13:**
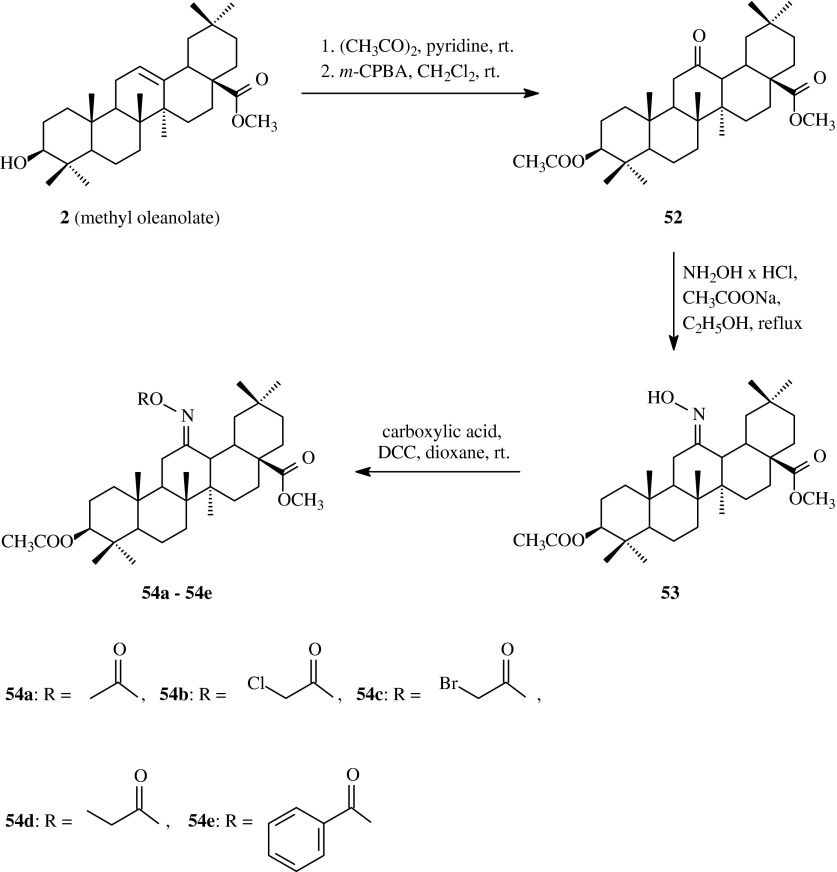
The synthesis of acylated oximes **54a–54e** derived from methyl oleanolate (**2**)

The other group of acylated oximes originated from oleanolic acid methyl ester (**2**) was obtained and described also by Bednarczyk-Cwynar et al. (2012b). It was the first described synthesis of acylated oximes within C ring of oleanane molecule (Scheme 13).

These new compounds were obtained as follows: first, methyl oleanolate (**2**) was acetylated with acetic anhydride in anhydrous pyridine according to literature protocol (Honda et al. 1997) and the resulted product was subjected to own-modified peracid oxidation (*m*-CPBA) in methylene chloride, based on literature data (Okamoto et al. 2000). After purification with column chromatography on SiO_2_ 12-oxocompound **52** was exposed to the action of hydroxylamine hydrochloride in ethanol according to a known method (Mukherjee et al. 2004). After that the resulted pure oxime **53** was acylated in dioxane with small excess of carboxylic acids in the presence of DCC at room temperature (Table 1, route 12). The resulting products **54a**–**54e** were obtained with the yields of above 90 %.

As the literature data showed (Soldi et al. 2008) the introduction of acyl group with short or slightly longer unbranched chain into the molecule of triterpene can improve the pharmacological activity of the new substance. On the basis of these results 12-ketone **52**, 12-oxime **53** and 12-acyloxyimino compounds **54a**–**54e** were subjected to the biological tests on order to estimate their cytotoxic activity (Bednarczyk-Cwynar et al. 2012b).

Oleanolic acid (**1**) as a reference compound and 12-ketone **52**, 12-oxime **53** as well as acyloxyiminoderivatives **54a**–**54e** were tested as to their cytotoxic activity against KB, MCF-7 and HeLa cell lines. Oleanolic acid (**1**) turned to be moderately active, with IC_50_ value for 14.93, 13.95 and 11.82 μM/l, respectively (Table 2). The introduction of propionoxyimino function at C-12 position resulted in significant intensification of anticancer activity, especially against KB cells. This derivative (**54d**) was about 21-fold more active against the above mentioned cell line (IC_50_ of 0.72 μM/l) than oleanolic acid (**1**), and about 6.5-fold more active against MCF-7 and HeLa cells (IC_50_ of 2.13 and 1.87 μM/l, respectively).

## Acyloxyimino derivatives of glycyrrhetinates

Simple acylated oximes are also known within glycyrrhetinates that are compounds with oleanane skeleton (Liu et al. 2007).

**Scheme 14:**
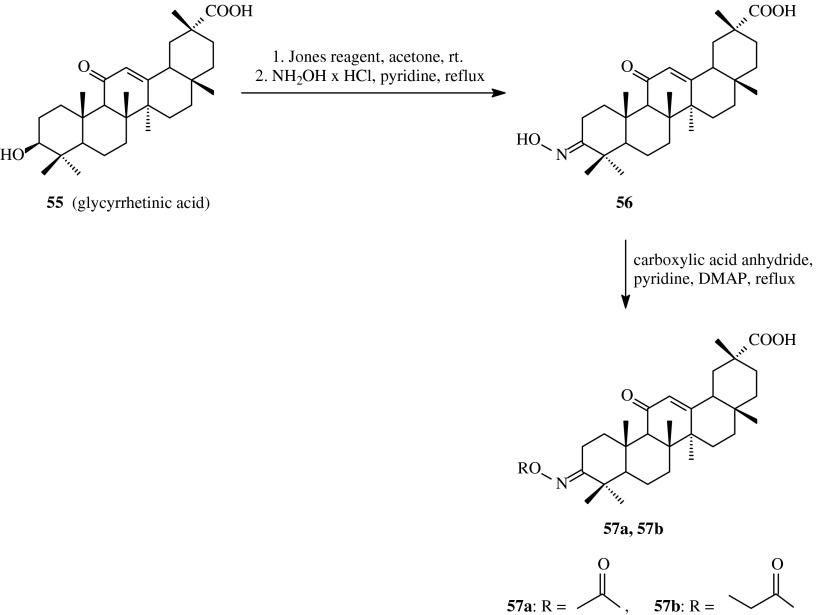
The synthesis of acylated oximes **57a**, **57b** derived from glycyrrhetinic acid (**55**)

Glycyrrhetinic acid (**55**) was oxidizied at C-3 position with the use of Jones reagent and next subjected to the action of hydroxylamine hydrochloride to give oxime **56** (Scheme 14). Next, the oxime **56** was transformed into its *O*-acyl derivatives upon the action of acetic or propionic anhydride in refluxed pyridine in the presence of catalytic amount of DMAP (Table 1, route 13). The crude products **57a** and **57b** were obtained with the yields of about 70 %.

Glycyrrhetinic acid (**55**) is a well known compound that inhibits growth and induces apoptosis of cancer cells. The antiproliferative and antiapoptotic effects for the above acid and its derivatives, e.g. acyloxyimino ones were determined with the use of human leukemia HL-60 cells.

The antiproliferative activity of glycyrrhetinic acid derivatives was determined by direct counting of leukemia cells which, as the first, were subjected to the action of triterpenes solutions and left for 3 days. To determine whether the antiproliferative effect of triterpenic derivatives was due to apoptosis induction, the percentage of apoptotic cells was determined after treatment of cells with the tested compounds. This value was for triterpenes **57a** and **57b** 19.1 and 27.7 %, respectively (Table 2). The GI_50_ values determined for compounds **57a** and **57b** were 58.8 and 57.7 μM, respectively, and they were slightly lower in comparison to mother glycyrrhetinic acid (GI_50_ = 63.2 μM) used as a reference substance (Liu et al. 2007).

## Summary

The increasing availability of oleanolic acid, betulin and glycyrrhetinic acids allows to obtain numerous derivatives of the above triterpenes. Among them the remarkable attention should be paid to highly promissing hydroxyimino triterpenes and the products of their acylation.

As the literature data show, the replacement of hydrogen atom within hydroxyimino function is one of the most effective method of the improvement of pharmacological activity of new triterpenic derivatives.

New derivatives of triterpenes, of the character of acyloximes, turned to be good antiviral agents, particularly against HIV, HSV-1 i H7N1 influenza, enterovirus ECHO-6, cytotoxic agents, e.g. against MOLT-4, JurkatE6.1, CEM.CM3, BRISTOL8, U937, DU145, PA-1, A549, L132 and HL-60 cell lines. Such compounds were also potent hepatoprotective agents, particularly for liver damage caused by the action of carbon tetrachloride and they decreased the antiulceration caused by the application of indomethacin or aspirin.

Cytotoxic and antiviral activity of the acylated triterpenic oximes can be considered as the most important and the most promissing type of biological activity.

The acylation reaction of the triterpenic oximes can be performed with the appliance of anhydrides in refluxed pyridine in the presence of DMAP, anhydrides in benzene in the presence of Et_3_N at room temperature, anhydrides in THF in the presence of DMAP at room temperature, anhydrides in pyridine at room temperature without a catalyst or activating agent, anhydrides in methylene chloride in the presence of Et_3_N and DMAP at room temperature, acid chlorides in DMF at room temperature without a catalyst or activating agent, acid chlorides in THF in 60 °C without a catalyst or activating agent, carboxylic acid in THF in the presence of DMAP and DCC at room temperature, as well as with carboxylic acid in dioxane in the presence of DCC at room temperature.

The most effective and the most convenient method for the synthesis of acylated triterpenic oximes turns out to be the last approach, as it proceeds to the highest yields of a desired product (over 90 %) with a high level of purity. This simple and quick manner involves small amounts of a solvent excellently soluble in water, that provides simple workup.
